# Stable nitrogen and carbon isotope compositions in plant-soil systems under different land-use types in a red soil region, Southeast China

**DOI:** 10.7717/peerj.13558

**Published:** 2022-06-06

**Authors:** Man Liu, Guilin Han

**Affiliations:** Institute of Earth Sciences, China University of Geosciences (Beijing), Beijing, China

**Keywords:** Soil organic nitrogen, δ15N composition, 15N enrichment factor, Land-use types, Red soil

## Abstract

**Background:**

Stable N isotope compositions in plant-soil systems have been widely used to indicate soil N transformation and translocation processes in ecosystems. However, soil N processes and nitrate (${\mathrm{NO}}_{3}^{-}$) loss potential under different land-use types are short of systematic comparison in the red soil region of Southeast China.

**Methods:**

In the present study, the stable N and C isotope compositions (*δ*^15^N and *δ*^13^C) of soil and leaf were analyzed to indicate soil N transformation processes, and the soil to plant ^15^N enrichment factor (*EF*) was used to compare soil ${\mathrm{NO}}_{3}^{-}$ loss potential under different land-use types, including an abandoned agricultural land, a natural pure forest without understory, and a natural pure forest with a simple understory.

**Results:**

The foliar *δ*^15^N value (−0.8‰) in the abandoned agricultural land was greater than those of the forest lands (ranged from −2.2‰ to −10.8‰). In the abandoned agricultural land, *δ*^15^N values of soil organic nitrogen (SON) increased from 0.8‰ to 5.7‰ and *δ*^13^C values of soil organic carbon (SOC) decreased from −22.7‰ to −25.9‰ with increasing soil depth from 0–70 cm, mainly resulting from SON mineralization, soil organic matter (SOM) decomposition, and C_4_ plant input. In the soils below 70 cm depth, *δ*^15^N values of SON (mean 4.9‰) were likely affected by microbial assimilation of ^15^N-depleted ${\mathrm{NO}}_{3}^{-}$. The variations in *δ*^15^N values of soil profiles under the two forests were similar, but the *EF* values were significant different between the pure forest with a simple understory (−10.0‰) and the forest without understory (−5.5‰).

**Conclusions:**

These results suggest that soil to plant ^15^N enrichment factor have a great promise to compare soil ${\mathrm{NO}}_{3}^{-}$ loss potential among different ecosystems.

## Introduction

Nitrogen (N) cycling is regarded as one of the most vital processes in terrestrial ecosystems, which is closely associated with vegetation growth and soil organic carbon (SOC) sequestration (Bae et al., 2015; Currie, Nadelhoffer & Aber, 2004; Galloway et al., 2008; Garten et al., 2007; Gogoi, Ahirwal & Sahoo, 2021; Kalinina et al., 2019; Li et al., 2017; Lin et al., 2019; Liu et al., 2017). However, since the Industrial Revolution, large exogenous N inputs, including the application of chemical N fertilizer and atmospheric N deposition, has caused N saturation in ecosystems in many tropical and subtropical regions (Galloway et al., 2008; Muhammed et al., 2018; Song et al., 2021). At present, many environmental problems, such as soil nitrate (${\mathrm{NO}}_{3}^{-}$) loss and N_2_O emissions are of great concern, because of the resulting N pollution to aquatic ecosystems and atmospheric ecosystems (Dai et al., 2020; Choi et al., 2017). Thus, research focused on N processes and N loss potential have important implications for environmental management.

With the wide application of stable isotope technology in earth surface environments (Li et al., 2022; Zeng et al., 2022), the stable N isotope ratio (^15^N/^14^N, δ^15^N) has been also widely applied to evaluate soil N cycling patterns in various ecosystems (Kayler et al., 2011; Lim et al., 2015; Pardo et al., 2007). Soil δ^15^N composition can be affected by different N sources and a series of transformation processes, such as symbiotic N fixation with mycorrhiza (Hobbie & Ouimette, 2009; Taylor, Chazdon & Menge, 2019), atmospheric N deposition (Currie, Nadelhoffer & Aber, 2004; Liu, Yeh & Sheu, 2006), plant uptake and microbial assimilation (Fowler et al., 2013; Waser et al., 1998), soil organic nitrogen (SON) mineralization (Liu et al., 2017; Zhang et al., 2015), nitrification and denitrification (Robinson, 2001; Galloway et al., 2008), and ammonia (NH_3_) volatilization (Choi et al., 2017). In agricultural ecosystems, there are many challenges to evaluate soil N dynamics using δ^15^N composition because the application of N-fertilizer affects soil N pool composition and N transformation processes, as well as δ^15^N abundance (Baggs et al., 2003; Jia et al., 2018; Zhu, Deng & Shangguan, 2018). Crop N is mainly derived from synthetic fertilizer and manure with significantly differing δ^15^N abundance of 0.3 ± 0.2‰ and 7.8 ± 0.6‰, respectively (Choi et al., 2005). These N fertilizers indirectly affect soil δ^15^N composition by straw turnover (Baggs et al., 2003; Corre et al., 2007). Furthermore, a series of N transformation and translocation processes in soils can cause substantial δ^15^N fractionation (Robinson, 2001). For example, applying manure with a low C/N ratio enhances the SON mineralization rate, which produces ^15^N-depleted ammonium (${\mathrm{NH}}_{4}^{+}$) and causes ^15^N enrichment in residual organic matter and microbes (Choi et al., 2017; Koopmans et al., 1997). Applying ${\mathrm{NH}}_{4}^{+}$ fertilizer accelerates nitrification and NH_3_ volatilization, which causes ^15^N enrichment in residual ${\mathrm{NH}}_{4}^{+}$ and produces ^15^N-depleted ${\mathrm{NO}}_{3}^{-}$ and NH_3_, respectively (Choi, Matushima & Ro, 2011). Applying ${\mathrm{NO}}_{3}^{-}$ fertilizer affects denitrification and increases ${\mathrm{NO}}_{3}^{-}$-N leaching loss. Denitrification causes ^15^N enrichment in residual ${\mathrm{NO}}_{3}^{-}$ and produces ^15^N-depleted NO_X_ and N_2_ (Choi et al., 2017). Although δ^15^N fractionation does not occur in the process of ${\mathrm{NO}}_{3}^{-}$-N leaching, the effect on the whole soil δ^15^N composition is significant (Corre et al., 2007). Microbial assimilation preferentially absorbs ${\mathrm{NH}}_{4}^{+}$ rather than ${\mathrm{NO}}_{3}^{-}$ due to the higher energy requirement in utilizing ${\mathrm{NO}}_{3}^{-}$ as an N source, except when the supply of ${\mathrm{NH}}_{4}^{+}$ is insufficient (Choi, Matushima & Ro, 2011). Thus, the application of N fertilizer alters the original soil N cycling patterns, as well as the δ^15^N composition of different N pools (Choi et al., 2017). Theoretically, soil N transformation processes can be inferred when the δ^15^N composition and the flux of N sources are known, and vice versa (Lim et al., 2015). However, it is incorrect to assume a single soil δ^15^N abundance to indicate soil N sources or processes in agricultural ecosystems (Kayler et al., 2011; Koopmans et al., 1997). In the present study, we used the soil δ^15^N and δ^13^C composition combined with the C/N ratio to provide more effective information about soil N sources and processes in agricultural land.

In forest ecosystems, the natural ^15^N-abundance of plants and organic soil horizons have been widely used to compare N saturation status at different sites within a basin (Boeckx et al., 2005; Koopmans et al., 1997; Shan et al., 2019). In many tropical and subtropical N-saturated forests, the δ^13^C compositions of plants and soils are gradually elevated along a gradient of increasing nitrification and ${\mathrm{NO}}_{3}^{-}$ loss (Li et al., 2017; Osborne et al., 2017; Ross, Lawrence & Fredriksen, 2004; Soper et al., 2018). The continuous loss of ^15^N-depleted ${\mathrm{NO}}_{3}^{-}$ leads to ^15^N enrichment in soils, ultimately causing ^15^N-enriched foliage by plant uptake (Kahmen, Wanek & Buchmann, 2008). However, the δ^13^C values of plants or soils do not always show excellent corresponding relationships with net nitrification rate and the quantity of ${\mathrm{NO}}_{3}^{-}$ loss (Pardo et al., 2007). Soil δ^13^C composition is affected by multiple N processes and litter δ^13^C abundance, and plant δ^13^C composition is affected by species and rooting depth (Liu, Yeh & Sheu, 2006; Pardo et al., 2007; Ross, Lawrence & Fredriksen, 2004). Thus, the absolute values of foliar or soil ^15^N-abundance alone are, sometimes, not adequate to compare the N status saturation at different sites within a basin. The soil to plant ^15^N enrichment factor (*EF*) greatly optimizes these deficiencies, in which the δ^15^N value of the surface soil is used to calibrate foliar ^15^N enrichment at specific sites (Garten & Van Miegroet, 1994; Garten et al., 2007; Pardo et al., 2007). In the present study, we further optimize the *EF* method to facilitate comparison of soil N loss potential under different land-use types within a basin.

In Southeast China, red soils cover 11.8% of the country’s land surface but provide a food supply for 22.5% of the population (Lin et al., 2012). The red soil is a typical acid soil that does not readily hold nutrients under strong leaching (Zhang et al., 2013), including available inorganic N. Considering the demand for crop production and cross-ecosystem N pollution, soil N transformation processes and ${\mathrm{NO}}_{3}^{-}$ loss potential are important topics of study in the red soil region. Furthermore, high levels of atmospheric N deposition occur in the subtropical red soil region, resulting in N-saturated ecosystems (Lin et al., 2012; Zhou et al., 2010). However, differences in soil N transformation processes and ${\mathrm{NO}}_{3}^{-}$ loss potential under different land-use types have not been systematically compared in this area. In the present study, we attempt to (1) compare δ^15^N and δ^13^C compositions, and SOC/SON ratios in soil profiles and analyze soil N processes under different land-use types; and (2) compare soil to plant ^15^N *EF* values and evaluate soil ${\mathrm{NO}}_{3}^{-}$ loss potentials under different land-use types. This research can improve understanding of soil N use efficiency under land-use types, which has important implications for environmental management in the red soil region of Southeast China.

**Figure 1:**
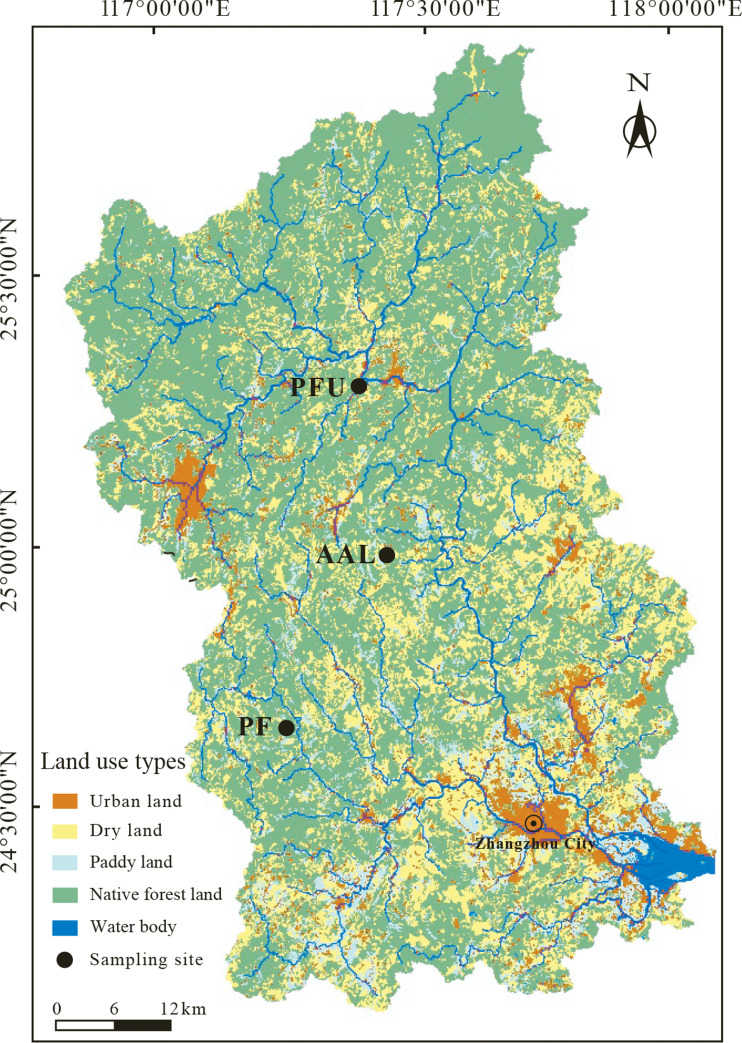
The distribution of land-use types in the Jiulongjiang River basin and the location of soil sampling sites. AAL, abandoned agricultural land; PF, natural pure forest without understory; PFU, natural pure forest with a simple understory.

## Materials and Methods

### Study area

The study area is located in the Jiulongjiang River basin (24°18′–25°88′N, 116°78′–118°03′E, Fig. 1) of Fujian province, Southeast China. This basin has a drainage area of 14,741 km^2^. The average elevation is less than 200 m (above sea level). The terrain of the basin transits from mountain to plain along a north to south transect (Liu & Han, 2021). The basin experiences a subtropical oceanic monsoon climate, with annual precipitation of 1,400–1,800 mm and annual temperature of 19.9–21.1 °C. In this humid climate, the red soils, classified as Ultisols in the soil taxonomy of the United States Department of Agriculture (USDA) (Soil Survey Staff, 2014), are widely developed in the basin. The red soils are strongly acid with a range of soil pH from 2.8 to 5.1 (Table 1). Furthermore, the soils mainly consist of silt-sized particles with a range of 54% to 77% (v/v%), sand-sized particles account for 5–31%, and clay-sized particles account for 7–22% (Table 1). The soils are silt loams, according to the soil taxonomy of the USDA (Soil Survey Staff, 2014). Native forests account for over 60% of the basin area, and other lands are used for agriculture and residential area (Liu & Han, 2021).

**Table 1:** Information about location, soil pH, soil texture, and land-use type of soil sampling sites.

Sampling site	Longitude and latitude	Elevation	Slope aspect and slope gradient	Soil pH	Soil texture and different-sized particle proportion	Land-use type and vegetation structure
AAL	117°30′9.49″E; 24°59′39.7″N	128 m	South-facing slope; <5°	3.8–4.7 (4.1 ± 0.3)	Silty loam Clay: 10–17% (13 ± 2%) Silt: 62–72% (68 ± 2%) Sand: 16–27% (19 ± 3%)	Abandoned agricultural land: the tea garden (*Camellia sinensis*, C_3_) has been abandoned for 6 years. Goose-grass (*Eleusine indica*, C_4_) sporadically grows on the ground now. Chemical N-fertilizer were applied during crop planting.
PF	117°14′5.16″E; 24°39′6.33″N	131 m	South-facing slope; <5°	4.3–5.1 (4.7 ± 0.1)	Silty loam Clay: 10–22% (16 ± 3%) Silt: 54–77% (65 ± 5%) Sand: 5–31% (20 ± 6%)	Natural pure forest without understory: tall sandalwood (*Dalbergia odorifera*, C_3_) almost entirely occupied the canopy without understory plants.
PFU	117°25′28.11″E; 25°16′21.08″N	221 m	South-facing slope; <5°	4.1–4.6 (4.3 ± 0.1)	Silty loam Clay: 7–14% (11 ± 1%) Silt: 59–69% (64 ± 2%) Sand: 19–31% (25 ± 3%)	Natural pure forest with a simple understory: pine (*Pinus tabuliformis*, C_3_) occupied the canopy and fern (*Pteridium aquilinum*, C_3_) occupied the understory.

**Notes.**

Soil pH and the proportion of different-sized particles are expressed as Minimum–Maximum (Mean ± SD).

### Sampling

According to the main land-use types in the basin (Fig. 1), the three sampling sites representing abandoned agricultural land (AAL), natural pure forest without understory (PF), and natural pure forest with a simple understory (PFU) were selected. The abandoned agricultural land at the AAL site underwent a conversion from tea garden (*Camellia sinensis*, C_3_ plant) to wasteland 6 years ago. During the tea planting period from 1985 to 2012, chemical N-fertilizer, including urea, ammonium sulfate, and ammonium bicarbonate, provided 45 kg N per year to support 100 kg tea production. Six years ago, the land was cleared of all tea trees. At present, the land is sporadically covered by goose-grass (*Eleusine indica*, C_4_ plant), and N-fertilizer is no longer applied. Average vegetation coverage of growing season at this site is approximately 45% and distribution of goose-grass roots focus on the depth of 0–10 cm. The pure forest at the PF site without understory consists of a single tree species, tall sandalwood (*Dalbergia odorifera*, C_3_ plant) trees occupy the canopy. Average vegetation coverage of growing season at this site is approximately 90% and 75% of underground biomass is in the layer of 0–30 cm. In contrast, the pure forest at the PFU site with a simple understory consists of two plant species, pines (*Pinus tabuliformis*, C_3_ plant) occupy the canopy, and ferns (*Pteridium aquilinum*, C_3_ plant) mainly occupy the understory (Table 1). Average vegetation coverage of growing season at this site is 100% and the roots of ferns and pines mainly distribute in the depth of 0–10 cm and 0–30 cm, respectively.

In January 2018, all sampling sites were selected at the section of building excavation and three parallel soil profiles were set up at each site. Considering the strong spatial heterogeneity of soil physicochemical properties in the horizontal and vertical direction, the average result of the parallel soil profiles easily causes incorrect information if the distance among the three parallel soil profiles is extremely distant. Thus, to fully consider both repeatability and representativeness of sampling sites, the distance between every two parallel soil profiles was set up in 50–100 m. A total of nine soil profiles with a thickness of 3 m were selected to collect soil samples. Soil samples were systematically collected from the bottom to the top of the profile at five cm-intervals. In each soil profile, three parallel samples with a horizontal distance of 1 meter were collected at each depth. To ensure representativeness of soil samples, three parallel samples were mixed to form a single sample.

Foliage samples were collected from the dominant vegetation near the soil profiles. The leaves of the dominant vegetation species were randomly collected without distinguishing between young and old leaves and mixed to form one sample. A total of three samples of goose-grass were collected at the AAL site, three samples of tall sandalwood were collected at the PF site, and three samples of pine and three samples of fern were collected at the PFU site.

### Sample analysis

Foliage samples were immediately preserved in a box filled with carbon dioxide ice and treated in the laboratory as quickly as possible. The foliage samples were washed with purified water to remove surface dust then freeze-dried and ground into powder (<75 µm). Soil samples were air-dried after removing gravel and fresh roots, then passed through a two mm sifter. Soil pH (soil/water: 1/2.5) was determined using a pH meter (Leici, Shanghai, China) with a precision of ± 0.05. For obtaining free soil mineral particles, soil samples (<2 mm) were digested with 10% hydrogen peroxide (H_2_O_2_) to remove organic bonding agents and with 2 mol/L hydrochloric acid (HCl) to remove calcareous cement, respectively (Yu et al., 2020; Liu, Han & Li, 2021a). Soil particle distributions were determined by a laser particle size analyzer (Mastersizer 2000, Malvern, England), with a precision of ± 1%. The sizes of soil particles were classified as: clay particles (<0.002 mm), silt particles (0.002–0.05 mm), and sand particles (0.05–2 mm) by Soil Survey Staff (2014).

Soil samples (<2 mm) were ground by an agate mortar and then passed through a 200-mesh (75 µm) sifter. For removing carbonates, inorganic N (mainly including ${\mathrm{NO}}_{3}^{-}$ and ${\mathrm{NH}}_{4}^{+}$), and dissolved organic carbon and nitrogen (DOC and DON), soil samples (<75 µm) were soaked in 0.5 mol/L HCl for 24 h (Midwood & Boutton, 1998) and in 2 mol/L potassium chloride (KCl) for 24 h (Meng, Ding & Cai, 2005), respectively. The treated samples were washed with purified water until neutrality, then dried in an oven at 55 °C until constant weight and ground into powder. The mass of samples before and after treatment was recorded. The foliar N content and SON content were measured by a multi-element analyzer (Vario TOC Cube, Elementar, Germany) in the Surficial Environment and Hydrological Geochemistry Laboratory, China University of Geosciences (Beijing). Standard soil substances (OAS B2152) were repeatedly measured to monitor the reproducibility. The precision of N content was better than ± 0.02%. The actual SON contents in the original soil samples were obtained after calibration by multiplying the measured value by the ratio of the sample mass after treatment to that before treatment (Liu, Han & Zhang, 2020).

The stable N isotope ratio (^15^N/^14^N) of SON and stable C isotope ratio (^13^C/^12^C) of SOC in soil and leaf samples were determined utilizing an isotope mass spectrometer (Thermo, MAT-253, USA) in the Center Laboratory for Physical and Chemical Analysis, Institute of Geographic Sciences and Natural Resources Research, Chinese Academy of Sciences. The measurements are expressed in standard δ notation (‰) to indicate the differences between the stable isotope ratio of the samples and accepted standard materials (atmospheric N_2_ and Vienna Pee Dee Belemnite (VPDB)), where:

(1) \begin{eqnarray*}& & {\mathrm{\delta }}^{15}{\mathrm{N}}_{\mathrm{ sample}}(\permil )=[({\mathrm{R}}_{\mathrm{sample}}-{\mathrm{R}}_{\mathrm{air}})/{\mathrm{R}}_{\mathrm{air}}]\times \text{1,000}, \mathrm{R}={}^{\text{15}}\mathrm{N}{/}^{\text{14}}\mathrm{N}\end{eqnarray*}

(2) \begin{eqnarray*}& & {\mathrm{\delta }}^{13}{\mathrm{C}}_{\mathrm{ sample}}(\permil )=[({\mathrm{R}}_{\mathrm{sample}}-{\mathrm{R}}_{\mathrm{V PDB}})/{\mathrm{R}}_{\mathrm{V PDB}}]\times \text{1,000}, \mathrm{R}={}^{\text{13}}\mathrm{C}{/}^{\text{12}}\mathrm{C}.\end{eqnarray*}

Standard substance (GBW04494, δ^15^N_Air_: −0.24‰  ± 0.13‰; δ^13^C_V PDB_: −45.6‰  ± 0.08‰) was used as reference material. The reproducibility was determined through replicate measurements of reference material, which was better than 0.1‰.

### Two end-member mixing model

Vegetation in the abandoned agricultural land (AAL site) suffered a conversion from tea tree (C_3_ plant) to goose-grass (C_4_ plant). The δ^15^N and δ^13^C values of SOM in soils mainly depend on the mixed results of these stable isotope compositions of organic matter derived from tea tree and goose-grass when δ^15^N and δ^13^C fractionations during SON mineralization and SOC decomposition processes are negligible or unconsidered. The contributions of organic matter derived from tea tree and goose-grass to total SON or SOC are calculated by the two end-member mixing model (Boutton et al., 1998; Guo et al., 2020; Liu, Han & Li, 2021b), as follows:

(3) \begin{eqnarray*}& & {\mathrm{\delta }}^{15}{\mathrm{N}}_{\mathrm{ sample}}={\mathrm{\delta }}^{15}{\mathrm{N}}_{\mathrm{ tea}}f+{\mathrm{\delta }}^{15}{\mathrm{N}}_{\mathrm{ grass}}(1-f)\end{eqnarray*}

(4) \begin{eqnarray*}& & {\mathrm{\delta }}^{13}{\mathrm{C}}_{\mathrm{ sample}}={\mathrm{\delta }}^{13}{\mathrm{C}}_{\mathrm{ tea}}f+{\mathrm{\delta }}^{13}{\mathrm{C}}_{\mathrm{ grass}}(1-f)\end{eqnarray*}

where δ^15^N_tea_ and δ^13^C_tea_ indicate the stable N and C isotope compositions of the end-member of tea tree source; δ^15^N_grass_ and δ^13^C_grass_ indicate the stable N and C isotope compositions of the end-member of goose-grass source. The *f* (%) is proportion of organic N or C derived from tea tree in total SON or SOC.

### Soil to plant ^15^N enrichment factor calculation

Plant N is mainly derived from available N in soils by uptake, thus plant ^15^N natural abundance is generally affected by the soil δ^15^N composition (Liu, Yeh & Sheu, 2006; Ross, Lawrence & Fredriksen, 2004). The soil to plant ^15^N enrichment factor (*EF*) is proposed to calibrate foliar ^15^N enrichment at the soil site with specific δ^15^N composition, the formula is shown as follows (Garten et al., 2007; Pardo et al., 2007):

(5) \begin{eqnarray*}& & EF={\mathrm{\delta }}^{15}{\mathrm{N}}_{\mathrm{ leaf}}-{\mathrm{\delta }}^{15}{\mathrm{N}}_{\mathrm{ soil}}\end{eqnarray*}

where δ^15^N_leaf_ is the δ^15^N composition of foliage samples of dominant species, δ^15^N_soil_ is determined by the δ^15^N composition in soils. However, it is not always clear what soil depth should be considered at a specific site. The principal soil layer where the fine roots are distributed is believed to provide most of the available N for plant uptake. Thus, the appropriate soil depth should depend on the root distribution of the corresponding plant, rather than a conventional definition such as 0–20 (or 30) cm. In this study, for the goose-grass at the AAL site and fern at the PFU site, the depth of soil was 0–10 cm; while for the sandalwood at the PF and pine at the PFU site, the depth of soil was 0–30 cm, as shown in Table 2. Generally, foliage δ^15^N values are less than those of soil δ^15^N (Baggs et al., 2003; Corre et al., 2007), thus the *EF* is typically a negative value. In an N-saturated ecosystem, the *EF* is positively correlated with net nitrification and ${\mathrm{NO}}_{3}^{-}$ loss (Garten & Van Miegroet, 1994). Thus, the *EF* can be employed to compare N cycling patterns at different sites within a basin (Pardo et al., 2007). When the *EF* approaches 0, high soil N loss potential at a site is indicated.

### Statistical analysis

Scatter plots of C/N ratios *vs* δ^15^N values and δ^13^C values *vs* δ^15^N values in the abandoned agricultural land at the AAL site were used to determine the distribution of soil samples relative to different end-members. Moreover, the relationships between them in the soils at the 0–80 cm depth were determined by the general linear model, and coefficients of *R*^2^ and *P*-value were exhibited. All statistical analyses were performed by SPSS 18.0 software (SPSS Inc., Chicago, IL, USA) and all figures were generated by SigmaPlot 12.5 software package (Systat Software GmbH, Erkrath, Germany) and Adobe Illustrator CS2 software (Adobe Systems Inc., San Jose, CA, USA).

## Results

### Distribution of SON contents and SOC/SON ratios in soil profiles

**Figure 2:**
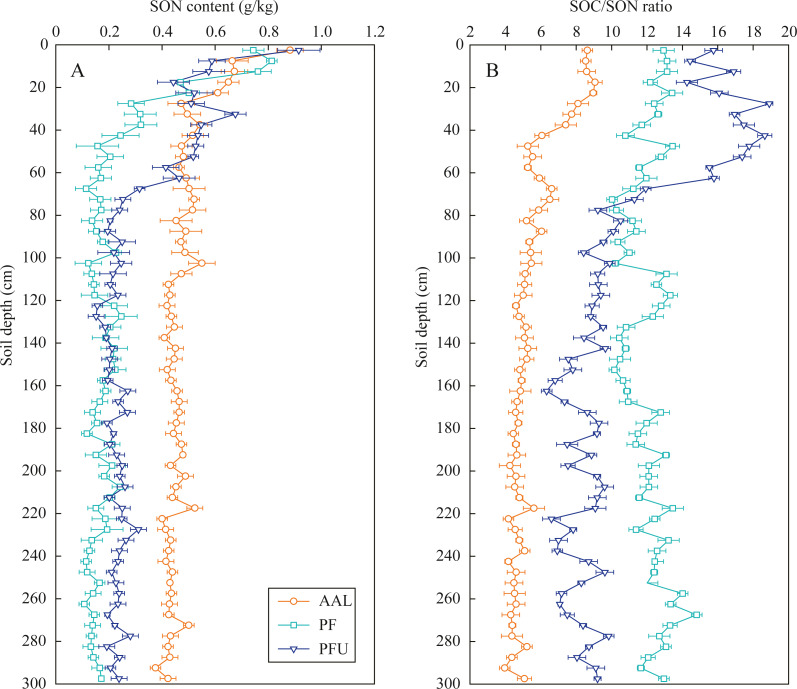
Distribution of SON content (A) and SOC/SON ratio (B) in soil profiles under different land-use types. Error bar is determined by standard error. AAL, abandoned agricultural land; PF, natural pure forest without understory; PFU, natural pure forest with a simple understory.

The SON contents in the three soil profiles at 0–60 cm depth intensively decreased with increasing soil depth, with a range from 0.88 g/kg to 0.44 g/kg at the AAL site, from 0.81 g/kg to 0.16 g/kg at the PF site, and from 0.91 g/kg to 0.25 g/kg at the PFU site (Fig. 2A). The SON contents in the three soil profiles below 60 cm depth slightly fluctuated, but they under the abandoned agricultural land (mean 0.45 g/kg) were 2–3 times greater than those under the forest land. Moreover, SON contents at the PFU site (mean 0.22 g/kg) were significantly greater than those at the PF site (mean 0.16 g/kg).

The SOC/SON ratios under the abandoned agricultural land slowly decreased from nine to four with increasing soil depth, and the ratios at all depths were the lowest among the three land-use types (Fig. 2B). In the forest lands, SOC/SON ratios at the PF site (10–13) were lower than those at the PFU site (10–16) in the 0–70 cm depth soils, while the ratios at the PF site (10–15) were higher than those at the PFU (6–10) site in the soils below the 70 cm.

### Distribution of δ^15^N and δ^13^C values in soil profiles

**Figure 3:**
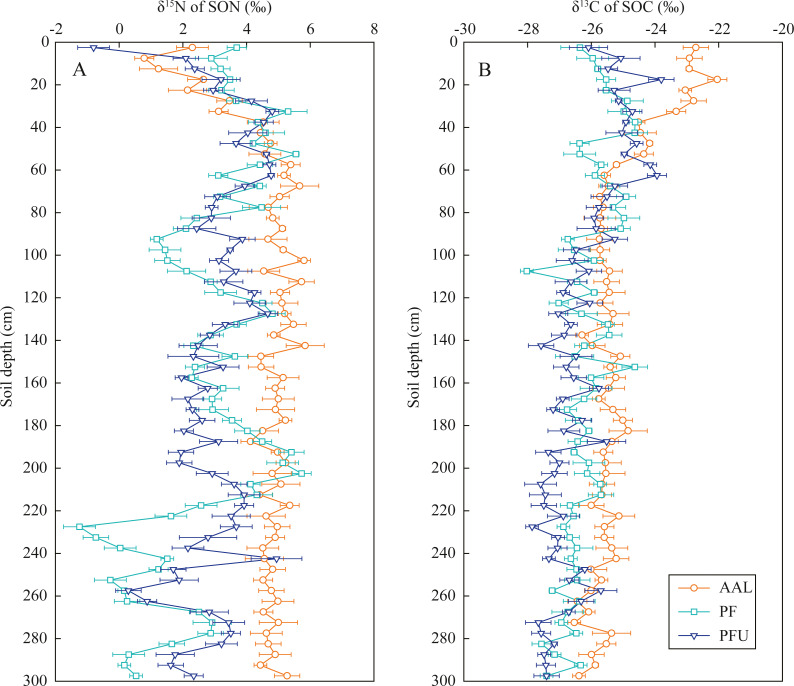
Distribution of *δ*^15^N values in SON (A) and *δ*^13^C values in SOC (B) in soil profiles under different land-use types. Error bar is determined by standard error. AAL, abandoned agricultural land; PF, natural pure forest without understory; PFU, natural pure forest with a simple understory.

In the abandoned agricultural land at the AAL site, the δ^15^N values of SON increased from 0.8‰  to 5.7‰ with increasing soil depth at the 0–70 cm depth and then slightly fluctuated near 4.9‰  below the 70 cm depth (Fig. 3A). In the forest land, the δ^15^N values in the soils at the 0–35 cm depth increased from 2.9‰  to 5.3‰  at the PF site and increased from −0.8‰  to 4.8‰  at the PFU site, with high variation in the soils below the 35 cm depth (mean 2.5‰ at the PF site and mean 2.8‰  at the PFU site).

In the soils at the 0–35 cm depth, the δ^13^C values of SOC increased with increasing soil depth at the PF site (from −26.4‰ to −24.6‰) and PFU (from −26.1‰ to −24.7‰), but decreased with increasing soil depth at the AAL site (from −22.7‰ to −23.3‰) (Fig. 3B). However, the δ^13^C values in the soils below the 35 cm depth at all sites showed slightly decreasing trends (generally by 1–2‰) with increasing soil depth (mean −25.6‰ at the AAL site, mean −26.4‰ at the PF site, and mean −26.8‰ at the PFU site).

### Soil to plant ^15^N *EF* under different land-use types

Soil to plant ^15^N *EF* was employed to compare N cycling patterns under different land-use types in the Jiulongjiang River basin (Table 2). The same *EF* value (−10.0‰) in the fern-soil and pine-soil systems at the PFU site provided confidence in applying the *EF* to pure forest with a simple understory. In tea plantation period, the input of chemical N-fertilizer significantly affects crop δ^15^N composition because it is the dominant source of crop N. Moreover, this effect can be further transmitted to the SON of surface soil through straw turnover and microbial assimilation. However, the available N absorbed by goose-grass (an annual herbage) in the abandoned agricultural land at the AAL site is mainly derived from SON mineralization and nitrification because N-fertilizer was not applied for 6 years. Thus, the *EF* is also applicable in the abandoned agricultural land in this study. We suggest that the *EF* can be employed to indicate N cycling in some special abandoned agricultural land, in which N-fertilizer is not applied or the application of N-fertilizer has been stopped for several years. The mean *EF* value at the PFU site (−10.0‰) was less than that at the PF site (−5.5‰), and much less than that at the AAL site (−3.1‰) (Table 2).

**Table 2:** Foliar δ^15^N and δ^13^C values, N and C contents and C/N ratios, soil δ^15^N value, and the soil to plant ^15^N enrichment factor (*EF*) at the three sites.

Site	Dominant vegetation	Foliar δ^15^N (‰)	Foliar δ^13^C (‰)	Foliar N content (%)	Foliar C content (%)	Foliar C/N ratio	Soil δ^15^N (‰)	*EF* value (‰)
AAL	Goose-grass (C_4_)	−0.8 ± 0.3	−14.2 ± 0.3	0.88 ± 0.21	52.1 ± 1.6	59.3 ± 1.2	2.3 ± 0.5	−3.1 ± 0.4
PF	Sandalwood (C_3_)	−2.2 ± 0.2	−30.9 ± 0.5	1.46 ± 0.13	52.7 ± 1.2	36.0 ± 0.8	3.4 ± 0.3	−5.5 ± 0.2
PFU	Fern (C_3_)	−10.8 ± 0.5	−28.8 ± 0.4	1.15 ± 0.09	53.3 ± 0.8	46.2 ± 1.3	−0.8 ± 0.3	−10.0 ± 0.4
	Pine (C_3_)	−7.3 ± 0.1	−28.6 ± 0.2	1.53 ± 0.12	58.7 ± 0.5	38.3 ± 0.9	2.7 ± 0.4	−10.0 ± 0.2

**Notes.**

The values are expressed as Mean ± SD; *EF* = δ^15^N_leaf_ − δ^15^N_soil_. Soil depth of goose-grass root is 0–10 cm (total samples, *n* = 6) at the AAL site; for, soil depth of main sandalwood root is 0–30 cm (total samples, *n* = 18) at the PF site; soil depths of fern root and main pine root are 0–10 cm (total samples, *n* = 6) and 0–30 cm (total samples, *n* = 18) at the PFU site.

AAL abandoned agricultural land PF natural pure forest without understory PFU natural pure forest with a simple understory

## Discussion

### Analyzation of soil N processes under different land-use types

**Figure 4:**
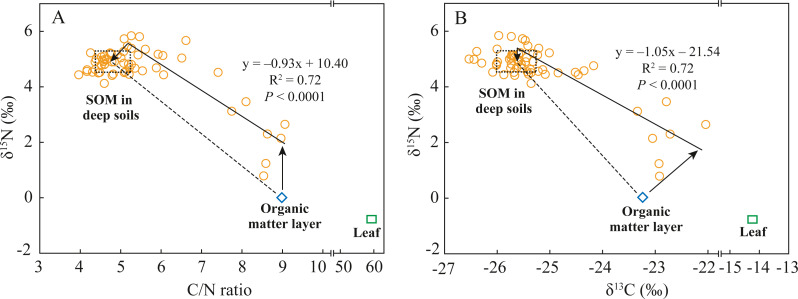
Relationships between C/N ratios and *δ*^15^N values (A) and between *δ*^13^C values and *δ*^15^N values (B) in soil and leaf under the abandoned agricultural land. The C/N ratios, *δ*^15^N values, and *δ*^13^C values in soil are SOC/SON ratios, *δ*^15^N values of SON, and *δ*^13^C values of SOC, respectively. The dashed rectangular box indicates the end-member values of C/N ratios, *δ*^15^N values, and *δ*^13^C values in the deep soils, which are determine by the Mean ± SD of these values. Dashed lines indicate the theoretical distribution region of these points in two end-member mixing model. Solid lines indicate the practical relationships of *δ*^15^N values with C/N ratio and *δ*^13^C values in soil, which are determined by the general linear model.

Soil N dynamics in abandoned agricultural lands are extremely complex because the interrupted application of chemical N-fertilizers changes the soil N pool composition and transformation processes (Baggs et al., 2003; Pardo et al., 2007). Soil N transformation processes can be identified by analyzing soil δ^15^N composition and stoichiometric SOC/SON ratio if N sources are known, and vice versa (Guo et al., 2020; Lim et al., 2015). In the abandoned agricultural land at the AAL site, the covered plant underwent a conversion from tea tree (C_3_) to goose-grass (C_4_) (Table 1). The average δ^13^C value of SOM in the deep soils was −25.6‰ (Fig. 4B), indicating the source of the C_3_ plant (tea tree). While the ^13^C-enriched (δ^13^C: −23.2‰) SOM in the organic matter layer was mainly attributed to the mixing of old SOM from past tea tree and new SOM from present goose-grass (δ^13^C: −14.2‰). According to the estimated results by a two end-member mixing model (Boutton et al., 1998), 94% of SOC in the surface soils was derived from past tea tree, while only 6% of SOC was derived from present goose-grass. The δ^15^N value of SON (0.01‰) in the organic matter layer was close to the foliar δ^15^N value of goose-grasses (−0.8‰), likely resulting from the effects of the application of chemical N-fertilizer (mean δ^15^N value: 0‰, Choi et al., 2017) during the tea plantation period. Long-term N-fertilizer application led to the δ^15^N value of tea tree and surface soil close to 0‰. The SON mineralization and nitrification in the surface soil provide available N (slightly lower than 0‰), which also affects the δ^15^N composition of goose-grass at present.

If the fractionation of δ^13^C and δ^15^N during SOM decomposition and soil N transformation processes were not considered, the distributions of δ^15^N values of SON, δ^13^C values of SOC, and SOC/SON ratios in the soils at the 0–80 cm depth conformed to the two end-member mixing model, as shown by the dashed lines in Fig. 4. The differences between the theoretical mixing line (dashed line) and the practical distribution line (solid line) could help to analyze soil N transformation processes in surface soils and deep soils. The δ^15^N value of SON and δ^13^C value of SOC in the organic matter layer were 2‰and 1.2‰ lower than those at the related end of the practical distribution line, respectively, while the SOC/SON ratios were almost the same (Fig. 4). In surface soils, organic matter decomposition generally causes a decrease in the SOC/SON ratio and causes ^13^C enrichment in SOC (Han et al., 2020). Goose-grass has a higher C/N ratio and δ^13^C value compared to the C_3_ plant (Table 2), thus δ^13^C value and SOC/SON ratio should increase with inputs of organic matter derived from C_4_ grasses. The difference in δ^13^C value is mainly attributed to SOM decomposition and C_4_ plant input. In surface soils, SON mineralization consumes organic N to increase SOC/SON ratio and causes ^15^N enrichment in organic residues (Choi et al., 2017; Koopmans et al., 1997). Thus, the difference in δ^15^N value is mainly attributed to SON mineralization. While no difference in SOC/SON ratios between them were likely a coincidental result of the combined influence of SOM decomposition, C_4_ plant input, and SON mineralization. The δ^15^N value of SON in the deep soils were 0.3‰ lower than those at the related end-member of the practical distribution line (Fig. 4). The difference in δ^15^N value of SON between them indicated that some N process cause ^15^N depletion of SON in deep soils. In the deep soils with an anoxic condition, denitrification and microbial assimilation of inorganic N are the two most common N processes (Baggs et al., 2003). However, microbial N (which is an important component of SON) is ^15^N-enriched during denitrification (Choi et al., 2017). Thus, it can be speculated that microbial assimilation of ^15^N-depleted inorganic N is likely the dominant N transformation process in deep soils.

Soil N processes in the forest ecosystems are less complex compared to those of the abandoned agricultural land. The δ^15^N values of SON and δ^13^C values of SOC in surface soils increased, while SOC/SON ratios decreased with increasing soil depth, these are closely associated with SON mineralization and SOM decomposition processes (Choi et al., 2017; Koopmans et al., 1997). The δ^15^N values of SON in the 0–20 cm layer at the PFU site were greater than those at the PF site (Fig. 3A), which is mainly affected by the δ^15^N composition of the above vegetation (Table 2). While the δ^15^N values were almost the same in the 20–80 cm layer at the two sites, indicating the same SON mineralization rate under pure forest.

**Figure 5:**
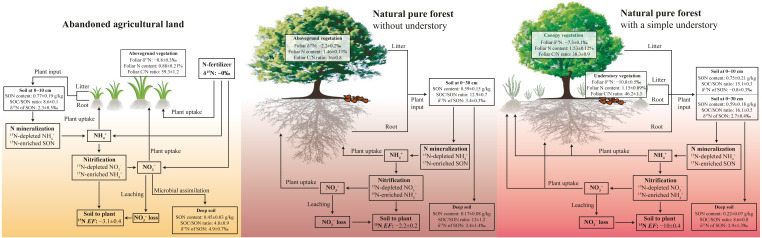
Conceptual figure of N processes in soil-plant system under different land-use types. The *EF* is soil to plant ^15^N enrichment factor, *EF* = *δ*^15^N_leaf_ − *δ*^15^N_soil_.

### Evaluation of soil N loss potential under different land-use types

In the pure forest with a simple understory at the PFU site, foliar δ^15^N of fern (−10.8‰) is less than that of pine (−7.3‰) (Table 2), likely associated with plant species and the depth of root distribution (Pardo et al., 2007). Foliar δ^15^N composition can differ between among plant species due to preferential absorption of ${\mathrm{NH}}_{4}^{+}$ or ${\mathrm{NO}}_{3}^{-}$ because ${\mathrm{NH}}_{4}^{+}$ is ^15^N-enriched compared to ${\mathrm{NO}}_{3}^{-}$ during nitrification (Choi et al., 2017; Currie, Nadelhoffer & Aber, 2004; Koopmans et al., 1997). Soil δ^15^N of SON increases with increasing soil depth, accordingly, available N produced from SON mineralization is also more positive with increasing soil depth (Han et al., 2020; Liu, Han & Li, 2021b). The influence of plant species and root depth on foliar δ^15^N will restrict the comparison of the N cycling patterns at different sites within a basin (Corre et al., 2007; Koopmans et al., 1997). However, our study proves that the soil to plant ^15^N *EF* is a more useful tool instead of foliar and soil δ^15^N in various and complex ecosystems. The *EF* value at the PFU site (−10.0‰) was less than that at the PF site (−5.5‰) (Table 2), indicating a lower ${\mathrm{NO}}_{3}^{-}$ loss potential in the pure forest land with a simple understory. Compared to the forest land without understory at the PF site, the additional ferns in the understory at the PFU site enhance the production of leaf litter, root exudates and residues, as well as plant uptake of available N (Fig. 5). The same soil net nitrogen mineralization rate indicates the same production rate of ${\mathrm{NH}}_{4}^{+}$ in soils under the two pure forests. However, more ${\mathrm{NH}}_{4}^{+}$ (and ${\mathrm{NO}}_{3}^{-}$) is absorbed by plants in the forest land with a simple understory, which results in less ${\mathrm{NH}}_{4}^{+}$ remaining in soils. The low ${\mathrm{NH}}_{4}^{+}$ content restricts nitrification to produce ${\mathrm{NO}}_{3}^{-}$, which means less ${\mathrm{NO}}_{3}^{-}$ loss. The *EF* value under abandoned agricultural land (−3.1‰) was greater than that under the two pure forests (Table 2), indicating a higher ${\mathrm{NO}}_{3}^{-}$ loss potential in abandoned agricultural land. Compared to the forest lands, ${\mathrm{NH}}_{4}^{+}$ in abandoned agricultural land is not sufficiently absorbed by the plant due to the lower root density. The high ${\mathrm{NH}}_{4}^{+}$ content promotes ${\mathrm{NO}}_{3}^{-}$ production by nitrification, which increases the risk of ${\mathrm{NO}}_{3}^{-}$ loss. Although soil to plant ^15^N *EF* can roughly indicates soil ${\mathrm{NO}}_{3}^{-}$ loss potential in different ecosystems, quantitative measurement of soil ${\mathrm{NO}}_{3}^{-}$ loss rate is needed to confirm *EF* result in future work.

## Conclusions

Soil N processes were analyzed and ${\mathrm{NO}}_{3}^{-}$ loss potential was estimated under different land-use types in the Jiulongjiang River basin. In the abandoned agricultural land, the δ^15^N values of leaf and SON record the signal from chemical N-fertilizer, even though fertilization has ceased for several years. The δ^15^N values of SON in the surface soils were mainly controlled by SON mineralization under all land-use types. The soil to plant *EF* value in the pure forest with a simple understory is greater than that in the pure forest without understory, indicating a lower ${\mathrm{NO}}_{3}^{-}$ loss potential. In the pure forest with a simple understory, plant species and the depth of root distribution affected foliar δ^15^N values of understory plants and canopy plants, but the *EF* value was not affected. The greatest *EF* value in the abandoned agricultural land indicated the highest ${\mathrm{NO}}_{3}^{-}$ loss potential compared to the two pure forests. These results suggest that soil to plant ^15^N *EF* have a great promise to indicate soil N loss potential in various ecosystems. But actual measurement of soil ${\mathrm{NO}}_{3}^{-}$ loss rate under different land-use types within a basin is needed to confirm *EF* result in future work.

## Supplemental Information

10.7717/peerj.13558/supp-1 Data S1 Raw data

## Additional Information and Declarations

### Data Availability

The following information was supplied regarding data availability: The raw measurements are available in the Supplementary File.

## References

[ref-1] Bae K, Fahey TJ, Yanai RD, Fisk M (2015). Soil nitrogen availability affects belowground carbon allocation and soil respiration in northern hardwood forests of New Hampshire. Ecosystems.

[ref-2] Baggs E, Stevenson M, Pihlatie M, Regar A, Cook H, Cadisch G (2003). Nitrous oxide emissions following application of residues and fertiliser under zero and conventional tillage. Plant and Soil.

[ref-3] Boeckx P, Paulino L, Oyarzun C, Van Cleemput O, Godoy R (2005). Soil δ^15^N patterns in old-growth forests of southern Chile as integrator for N-cycling. Isotopes in Environmental and Health Studies.

[ref-4] Boutton T, Archer SR, Midwood AJ, Zitzer SF, Bol R (1998). δ^13^C values of soil organic carbon and their use in documenting vegetation change in a subtropical savanna ecosystem. Geoderma.

[ref-5] Choi WJ, Chang SX, Allen HL, Kelting DL, Ro HM (2005). Irrigation and fertilization effects on foliar and soil carbon and nitrogen isotope ratios in a loblolly pine stand. Forest Ecology and Management.

[ref-6] Choi WJ, Kwak JH, Lim SS, Park HJ, Chang SX, Lee SM, Arshad MA, Yun SI, Kim HY (2017). Synthetic fertilizer and livestock manure differently affect δ^15^N in the agricultural landscape: a review. Agriculture, Ecosystems and Environment.

[ref-7] Choi WJ, Matushima M, Ro HM (2011). Sensitivity of soil CO_2_ emissions to fertilizer nitrogen species: urea, ammonium sulfate, potassium nitrate, and ammonium nitrate. Journal of the Korean Society for Applied Biological Chemistry.

[ref-8] Corre MD, Brumme R, Veldkamp E, Beese F (2007). Changes in nitrogen cycling and retention processes in soils under spruce forests along a nitrogen enrichment gradient in Germany. Global Change Biology.

[ref-9] Currie WS, Nadelhoffer KJ, Aber JD (2004). Redistributions of highlight turnover and replenishment of mineral soil organic N as a long-term control on forest C balance. Forest Ecology and Management.

[ref-10] Dai W, Bai E, Li W, Jiang P, Zheng X (2020). Predicting plant-soil N cycling and soil N_2_O emissions in a Chinese old-growth temperate forest under global changes: uncertainty and implications. Soil Ecology Letters.

[ref-11] Fowler D, Pyle JA, Raven JA, Sutton MA (2013). The global nitrogen cycle in the twenty-first century: introduction. Philosophical Transactions of the Royal Society of London. Series B.

[ref-12] Galloway JN, Townsend AR, Erisman JW, Bekunda M, Cai Z, Freney JR, Martinelli LA, Seitzinger SP, Sutton MA (2008). Transformation of the nitrogen cycle: recent trends, questions, and potential solutions. Science.

[ref-13] Garten CT, Hanson PJ, Todd DE, Lu BB, Brice DJ, Michener R, Lajtha K (2007). Natural ^15^N- and ^13^C-abundance as indicators of forest nitrogen status and soil carbon dynamics. Stable isotopes in ecology and environmental.

[ref-14] Garten CT, Van Miegroet HM (1994). Relationships between soil nitrogen dynamics and natural ^15^N-abundance in plant foliage from the Great Smoky Mountains National Park. Canadian Journal of Forest Research.

[ref-15] Gogoi A, Ahirwal J, Sahoo UK (2021). Evaluation of ecosystem carbon storage in major forest types of Eastern Himalaya: implications for carbon sink management. Journal of Environmental Management.

[ref-16] Guo Q, Wang C, Wei R, Zhu G, Cui M, Okolic CP (2020). Qualitative and quantitative analysis of source for organic carbon and nitrogen in sediments of rivers and lakes based on stable isotopes. Ecotoxicology and Environmental Safety.

[ref-17] Han G, Tang Y, Liu M, Van Zwieten L, Yang X, Yu C, Wang H, Song Z (2020). Carbon-nitrogen isotope coupling of soil organic matter in a karst region under land use change, Southwest China. Agriculture, Ecosystems and Environment.

[ref-18] Hobbie EA, Ouimette AP (2009). Controls of nitrogen isotope patterns in soil profiles. Biogeochemistry.

[ref-19] Jia X, Zhu Y, Huang L, Wei X, Fang Y, Wu L, Binley A, Shao M (2018). Mineral N stock and nitrate accumulation in the 50–200 m profile on the Loess Plateau. Science of the Total Environment.

[ref-20] Kahmen A, Wanek W, Buchmann N (2008). Foliar δ^15^N values characterize soil N cycling and reflect nitrate or ammonium preference of plants along a temperate grassland gradient. Oecologia.

[ref-21] Kalinina O, Cherkinsky A, Chertov O, Goryachkin S, Kurganova I, Lopes de Gerenyu V, Lyuri D, Kuzyakov Y, Giani L (2019). Post-agricultural restoration: implications for dynamics of soil organic matter pools. Catena.

[ref-22] Kayler ZE, Kaiser M, Gessler A, Ellerbrock RH, Sommer M (2011). Application of δ^13^C and δ^15^N isotopic signatures of organic matter fractions sequentially separated from adjacent arable and forest soils to identify carbon stabilization mechanisms. Biogeosciences.

[ref-23] Koopmans CJ, Dam DV, Tietema A, Verstraten JM (1997). Natural ^15^N abundance in two nitrogen saturated forest ecosystems. Oecologia.

[ref-24] Li X, Han G, Liu M, Liu J, Zhang Q, Qu R (2022). Potassium and its isotope behaviour during chemical weathering in a tropical catchment affected by evaporite dissolution. Geochimica et Cosmochimica Acta.

[ref-25] Li D, Yang Y, Chen H, Xiao K, Song T, Wang K (2017). Soil gross nitrogen transformations in typical karst and nonkarst forests, southwest China. Journal of Geophysical Research: Biogeosciences.

[ref-26] Lim SS, Kwak JH, Lee KS, Chang SX, Yoon KS, Kim HY, Choi WJ (2015). Soil and plant nitrogen pools in paddy and upland ecosystems have contrasting δ^15^N. Biology and Fertility of Soils.

[ref-27] Lin S, Iqbal J, Hu R, Ruan L, Wu J, Zhao J, Wang P (2012). Differences in nitrous oxide fluxes from red soil under different land uses in mid-subtropical China. Agriculture, Ecosystems and Environment.

[ref-28] Lin Y, Slessarev EW, Yehl ST, D’Antonio CM, King JY (2019). Long-term nutrient fertilization increased soil carbon storage in California grasslands. Ecosystems.

[ref-29] Liu M, Han G (2021). Distribution of soil nutrients and erodibility factor under different soil types in an erosion region of Southeast China. PeerJ.

[ref-30] Liu M, Han G, Li X (2021a). Contributions of soil erosion and decomposition to SOC loss during a short-term paddy land abandonment in Northeast Thailand. Agriculture, Ecosystems and Environment.

[ref-31] Liu M, Han G, Li X (2021b). Using stable nitrogen isotope to indicate soil nitrogen dynamics under agricultural soil erosion in the Mun River basin, Northeast Thailand. Ecological Indicators.

[ref-32] Liu M, Han G, Zhang Q (2020). Effects of agricultural abandonment on soil aggregation, soil organic carbon storage and stabilization: results from observation in a small karst catchment, Southwest China. Agriculture, Ecosystems and Environment.

[ref-33] Liu Y, Wang C, He N, Wen X, Gao Y, Li S, Niu S, Butterbach-Bahl K, Luo Y, Yu G (2017). A global synthesis of the rate and temperature sensitivity of soil nitrogen mineralization: latitudinal patterns and mechanisms. Global Change Biology.

[ref-34] Liu CP, Yeh HW, Sheu BH (2006). N isotopes and N cycle in a 35-year-old plantation of the Guandaushi subtropical forest ecosystem, central Taiwan. Forest Ecology and Management.

[ref-35] Meng L, Ding W, Cai Z (2005). Long-term application of organic manure and nitrogen fertilizer on N_2_O emissions, soil quality and crop production in a sandy loam soil. Soil Biology and Biochemistry.

[ref-36] Midwood AJ, Boutton TW (1998). Soil carbonate decomposition by acid has little effect on δ^13^C of organic matter. Soil Biology and Biochemistry.

[ref-37] Muhammed SE, Coleman K, Wu L, Bell VA, Davies JAC, Quinton JN, Carnell EJ, Tomlinson SJ, Dore AJ, Dragosits U, Naden PS, Glendining MJ, Tipping E, Whitmore AP (2018). Impact of two centuries of intensive agriculture on soil carbon, nitrogen and phosphorus cycling in the UK. Science of the Total Environment.

[ref-38] Osborne BB, Nasto MK, Asner GP, Balzotti CS, Cleveland CC, Sullivan BW, Taylor PG, Townsend AR, Porder S (2017). Climate, topography, and canopy chemistry exert hierarchical control over soil N cycling in a neotropical lowland forest. Ecosystems.

[ref-39] Pardo LH, Hemond HF, Montoya JP, Pett-Ridge J (2007). Natural abundance ^15^N in soil and litter across a nitrate-output gradient in New Hampshire. Forest Ecology and Management.

[ref-40] Robinson D (2001). δ^15^N as an integrator of the nitrogen cycle. Trends in Ecology and Evolution.

[ref-41] Ross DS, Lawrence GB, Fredriksen G (2004). Mineralization and nitrification patterns at eight northeastern USA forested research sites. Forest Ecology and Management.

[ref-42] Shan Y, Huang M, Suo L, Zhao X, Wu L (2019). Composition and variation of soil δ^15^N stable isotope in natural ecosystems. Catena.

[ref-43] Soil Survey Staff (2014). Keys to soil taxonomy.

[ref-44] Song W, Liu XY, Hu CC, Chen GY, Liu XJ, Walters WW, Michalski G, Liu CQ (2021). Important contributions of non-fossil fuel nitrogen oxides emissions. Nature Communications.

[ref-45] Soper FM, Taylor PG, Wieder WR, Weintraub SR, Cleveland CC, Porder S, Townsend AR (2018). Modest gaseous nitrogen losses point to conservative nitrogen cycling in a lowland tropical forest watershed. Ecosystems.

[ref-46] Taylor BN, Chazdon RL, Menge DNL (2019). Successional dynamics of nitrogen fixation and forest growth in regenerating Costa Rican rainforests. Ecology.

[ref-47] Waser NAD, Harrison PJ, Nielsen B, Calvert SE, Turpin DH (1998). Nitrogen isotope fractionation during the uptake and assimilation of nitrate, nitrite, ammonium, and urea by a marine diatom. Limnology and Oceanography.

[ref-48] Yu X, Zhou W, Wang Y, Cheng P, Hou Y, Xiong X, Du H, Yang L, Wang Y (2020). Effects of land use and cultivation time on soil organic and inorganic carbon storage in deep soils. Journal of Geographical Sciences.

[ref-49] Zeng J, Han G, Zhang S, Liang B, Qu R, Liu M, Liu J (2022). Potentially toxic elements in cascade dams-influenced river originated from Tibetan Plateau. Environmental Research.

[ref-50] Zhang X, Li Z, Tang Z, Zeng G, Huang J, Guo W, Chen X, Hirsh A (2013). Effects of water erosion on the redistribution of soil organic carbon in the hilly red soil region of southern China. Geomorphology.

[ref-51] Zhang Y, Zhang J, Zhu T, Mueller C, Cai Z (2015). Effect of orchard age on soil nitrogen transformation in subtropical China and implications. Journal of Environmental Sciences.

[ref-52] Zhou J, Cui J, Fan JL, Liang JN, Wang TJ (2010). Dry deposition velocity of atmospheric nitrogen in a typical red soil agro-ecosystem in Southeastern China. Environmental Monitoring and Assessment.

[ref-53] Zhu G, Deng L, Shangguan Z (2018). Effects of soil aggregate stability on soil N following land use changes under erodible environment. Agriculture, Ecosystems and Environment.

